# Recent advances on the roles of LncRNAs in cardiovascular disease

**DOI:** 10.1111/jcmm.15880

**Published:** 2020-09-24

**Authors:** Yexian Fang, Yuerong Xu, Runze Wang, Lang Hu, Dong Guo, Feng Xue, Wangang Guo, Dongwei Zhang, Jianqiang Hu, Yan Li, Wei Zhang, Mingming Zhang

**Affiliations:** ^1^ Department of Cardiology Tangdu Hospital The Fourth Military Medical University Xi’an Shaanxi China; ^2^ Department of Orthodontics School of Stomatology The Fourth Military Medical University Xi’an Shaanxi China

**Keywords:** biological function, cardiovascular diseases, Long Non‐coding RNA

## Abstract

Cardiovascular diseases are a main cause of mortality whose prevalence continues to increase worldwide. Long non‐coding RNAs (lncRNAs) regulate a variety of biological processes by modifying and regulating transcription of coding genes, directly binding to proteins and even coding proteins themselves. LncRNAs play key roles in the occurrence and development of myocardial infarction, heart failure, myocardial hypertrophy, arrhythmias and other pathological processes that significantly affect the prognosis and survival of patients with cardiovascular diseases. We here review the latest research on lncRNAs in cardiovascular diseases as a basis to formulate future research on prevention and treatment of cardiovascular diseases.

## 1 INTRODUCTION

The prevalence rate of cardiovascular diseases around the world continues to rise with the ageing of the population and changes in lifestyle.^1^ Cardiovascular disease ranks first among the causes of death among urban and rural residents in China.^2^ Non‐coding RNAs (ncRNAs) were originally thought as the ‘noise’ of protein‐coding gene transcription; however, protein‐coding genes account for less than 3% of the human genome and the importance of non‐coding RNAs in cardiovascular diseases is gradually being unravelled.^3^ Long non‐coding RNAs (lncRNAs), which are defined as non‐coding RNAs longer than 200 nucleotides, participate in biological individual growth and development, differentiation, apoptosis and other biological processes through diverse kinds of mechanisms which influence the occurrence and development of cardiovascular diseases.^4^ With progress in sequencing technology, new lncRNAs have been recognized as well as their various functions. In view of the relevance of lncRNAs in targeted prevention and treatment of cardiovascular diseases, this paper describes the latest insights on the roles of lncRNAs in cardiovascular diseases.

## 2 OVERVIEW OF LNCRNAS

LncRNAs were thought to be non‐functional by‐products of gene transcription. However, it has been proved that lncRNAs regulate gene expression through a variety of mechanisms, including affecting the stability of mRNAs and translation initiation, acting as a competitive endogenous RNA and regulating post‐translational modification, among other functions.^4^ Although lncRNAs cannot encode functional proteins, some might have coding ability, and the short peptides they encode may also be involved in the regulation of gene expression, then affecting the occurrence and development of diseases. For example, muscle‐specific LncRNA DOWRF encodes a short 34‐aminoacid peptide which regulates cardiac contractile function in mice by competitively combining with SERCA (sarcoplasmic reticulum calcium ATP enzyme).^5^

LncRNAs can be divided into antisense lncRNAs, bidirectional lncRNAs, intron lncRNAs and intergenic lncRNAs according to the position of lncRNAs in the genome.^6^ However, the limitation of this classification method has been highlighted by research findings and the growing number of discovered lncRNAs, which led some researchers to propose a new classification, which divides lncRNAs into eight broader categories: divergent, convergent, intronic, intergenic, overlapping sense, overlapping antisense, enhancer RNA and miRNA host gene.^7^, ^8^ However, the classification of lncRNAs leaves room for improvement, which will come from new insights on their function and mechanism.

## 3 BIOLOGICAL FUNCTIONS OF LNCRNAS

Cells under stress in the cardiovascular system may undergo apoptosis, autophagy, necrosis, fibrosis, proliferation or migration, leading to the occurrence and development of cardiovascular diseases. The biological functions of LncRNAs can be classified into four categories:

### 3.1 The lncRNA‐miRNA‐mRNA axis

MiRNAs inhibit the translation or promote the degradation of mRNAs by binding to the complementary sequence of their 3′ untranslated‐untranslated region (3′‐UTR), thus regulating gene expression,^9^ while lncRNAs can regulate the expression of mRNAs by acting as miRNAs sponges to interact with miRNAs.^10^

For example, Lu et al demonstrated that overexpression of lncRNA LOC100129973 improves the viability of endothelial cells, while its inhibition promotes endothelial cell apoptosis.^11^ Mechanistically, LOC100129973, as a miRNA sponge, competitively combines with miR‐4707‐5p and miR‐4767 to down‐regulate their RNA level. MiR‐4707‐5p and miR‐4767 are pro‐apoptotic molecules that down‐regulate the mRNA and protein levels of API5 (apoptosis inhibitor 5) and BCL2L12 (B‐cell lymphoma‐like protein 12), respectively. Therefore, lncRNA LOC100129973 has an anti‐apoptotic effect through lncRNA‐miRNA‐mRNA axis, which protects vascular endothelium. Meanwhile, lncRNA AK088388 was proved to regulate autophagy of cardiomyocytes by acting as an endogenous RNA sponge of miR‐30a during hypoxia/reoxygenation.^12^ Inhibition of AK088388 significantly reduces autophagy and cardiomyocyte injury by increasing miR‐30a and subsequently decreasing Beclin‐1 and LC3‐II.

### 3.2 Paracrine effects of lncRNAs

Cells in multicellular organisms communicate with nearby and remote cells through complex processes, which include paracrine signals and interactions with the extracellular matrix.^13^ Normal cardiovascular function requires coordination and communication among smooth muscle cells, endothelial cells, fibroblasts, immune cells and cardiomyocytes. Exosomes play a crucial role in cell‐to‐cell communication by transmitting various signal molecules (including proteins, mRNAs and non‐coding RNAs).^14^, ^15^

It was reported that exosomes derived from cardiac cells contain abundant non‐coding RNAs, which significantly promote the repair of ischaemic heart and angiogenesis and inhibit apoptosis.^16^ Zhu et al confirmed that exosomes derived from human UMSC (umbilical cord mesenchymal stem cells) prevent cardiac dysfunction caused by ageing, and the beneficial effect was attributed to exosomes/lncRNA MALAT1 (metastatic associated lung adenocarcinoma transcript 1)/NF‐κB/TNF‐ α pathway.^17^ In another study, exosomes derived from bone marrow mesenchymal stem cells (MSCs) treated with macrophage migration inhibitory factor (MIF) were shown to inhibit cardiomyocyte apoptosis induced by H_2_O_2_.^18^ Further study on the mechanism revealed that the beneficial effect was mediated by exosomes/lncRNA NEAT1/miR‐142‐3p/FOXO1 pathway. Therefore, exosome‐mediated therapy has potential for clinical application, and stem cells and exosomes are readily available. It should be pointed out that although exosomes obtained from stem cells in vitro protect the heart from injury and promote repair, the role of exosomes secreted by endogenous cardiomyocytes in the progression of heart disease remains unclear. More studies are needed to clarify mechanisms affecting exosome sources and their paracrine effects in cardiovascular diseases.^19^

### 3.3 Cis‐ and trans‐transcriptional regulation of lncRNAs

LncRNAs not only regulate neighbouring genes in cis‐conformation, but also play a role in trans‐regulation of expression of genes that are not closely related to their location.^20^ In other words, the subcellular localization of lncRNAs is crucial to their function. LncRNAs in nucleus can reprogramme gene expression, act as molecular scaffolds and activate or inhibit transcription by RNA‐DNA complexes.^21^ In contrast, lncRNAs enriched in the cytoplasm affect protein localization and regulate the stability and translation of mRNAs.^22^, ^23^

The most recognized example of lncRNAs in cis‐conformation is Xist. One of the X chromosomes in female mammals is transcriptionally silent during early embryonic development. Xist will make its sited X chromosome to relocate to the edge of the nucleus, inhibit the deposition of chromatin markers and eventually transcriptionally silence almost the entire chromosome through a series of events after being induced.^23^ In addition to lncRNAs regulating cis gene expression and chromatin status, more lncRNAs will regulate the transcription site and function in a trans‐regulated way. For example, the relationship between ANRIL and cardiovascular diseases susceptibility is associated with its ability to regulate trans gene expression, and Alu elements in ANRIL regulate the process of atherosclerosis through trans‐regulation of gene networks.^24^

### 3.4 LncRNAs interact with proteins directly

In addition to affecting the expression level of proteins by regulating transcription, lncRNAs can also interact with proteins to regulate their functions directly. Lee et al proved that lncRNA NORAD, which is widely and abundantly expressed in mammals, binds to PUMILIO directly to maintain genome stability.^25^ Moreover, there are molecules essential for mitosis, DNA repair and DNA replication among the interaction sites of PUMILIO. These molecules will be over‐suppressed, and therefore, chromosome segregation will be disrupted in the absence of NORAD. Another study documented that the expression of lncRNA ZFAS1 in cytoplasm and sarcoplasmic reticulum (SR) increased significantly in mice with myocardial infarction caused by coronary artery occlusion, and in a cell model of hypoxia; lncR‐ZFAS1 binds to SERCA2a (Ca^2+^ATP enzyme 2a of sarcoplasmic reticulum) directly and inhibits the activity of it in molecular level.^26^

**FIGURE 1:**
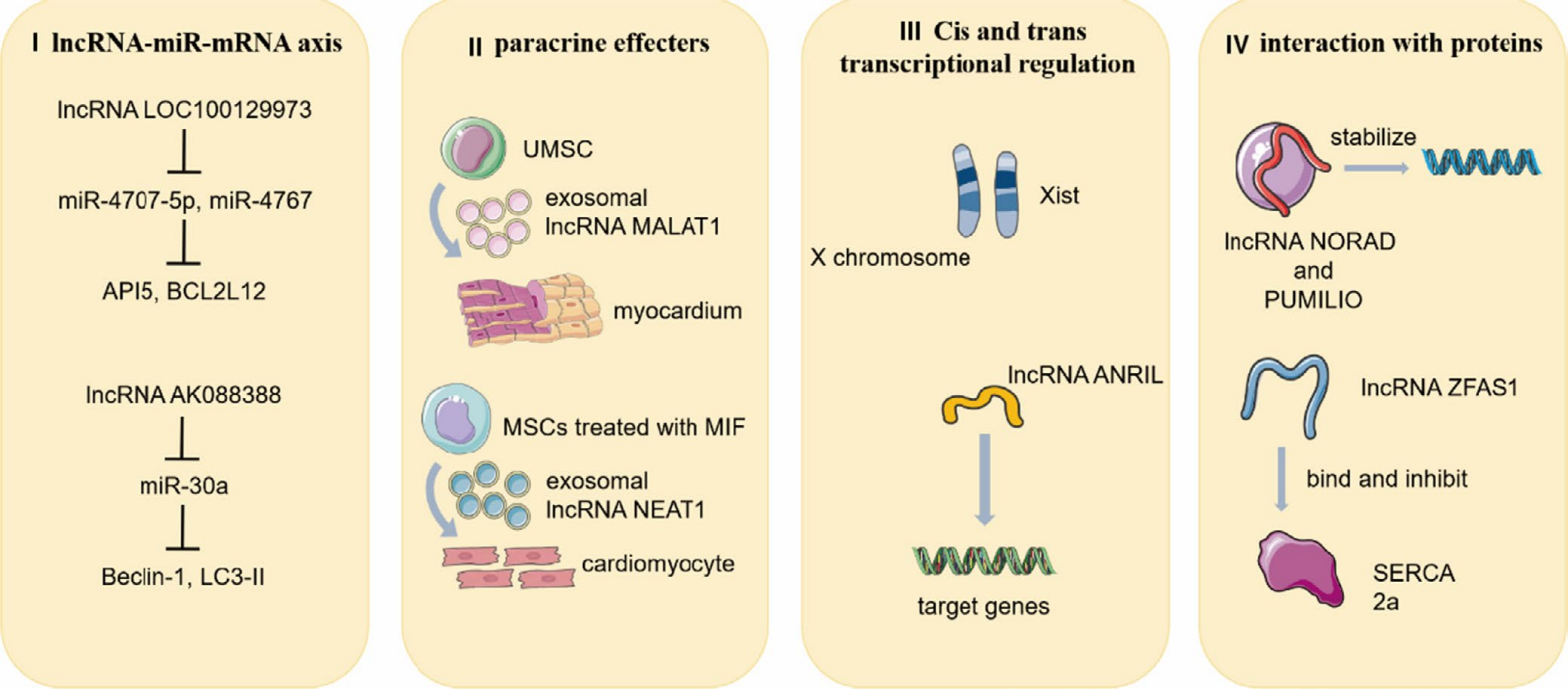
The biological functions of lncRNAs (I) LncRNA LOC100129973 can respectively affect the translation of API5 and BDL2L 12 through miR‐4707‐5p and miR‐4767; LncRNA AK088388 regulates Beclin‐1 and LC3‐II through miR‐30a. (II) LncRNAs that are transmitted by exosomes derived from stem cells can regulate the function of cardiomyocytes. (III) LncRNA Xist regulates its sited X chromosome in cis‐conformation while lncRNA ANRIL control target genes in a trans‐regulated way. (IV) LncRNA NORAD binds directly with PUMILIO to maintain the stability of the genome, and lncRNA ZFAS1 can bind and inhibit the activity of SERCA2a

LncRNAs perform a variety of biological functions in organisms not only through the lncRNA‐miRNA‐mRNA axis, but also by binding to proteins directly. In addition, lncRNAs participate in cis‐ and trans‐transcriptional regulation and play an important role in intercellular communication (see Figure 1).

## 4 LNCRNAS AND CARDIOVASCULAR DISEASES

### 4.1 LncRNAs and atherosclerosis

Atherosclerosis (AS), a chronic inflammatory change of blood vessels, is characterized by lipid necrosis and vascular wall sclerosis manifested as lipid deposition and smooth muscle cell proliferation in the intima of the artery. LncRNAs play significant parts in the apoptosis and autophagy of vascular endothelial cells, proliferation of smooth muscle cells, formation of foam cells, lipid metabolism and other processes, which are closely connected with AS.^27^ The genetic locus of 9p21.3 is considered to be the most influential common genetic risk factor for CAD (coronary heart disease), accounting for 10% to 15% of CAD in non‐African populations.^28^ LncRNA ANRIL, located at the gene region of chromosome 9p21, is increased in atherosclerotic plaques of patients with AS; increasing plaque size is related to the degree of atherosclerosis. LncRNA ANRIL is deemed to be the most reliable predictor of atherosclerotic risk and cardiovascular events.^4^, ^29^ In addition to being biomarkers, lncRNAs influence the occurrence and progression of AS through the following mechanisms:

#### 4.1.1 Regulation of cellular immunity

Endothelial cell dysfunction is an important cause of AS, and eNOS is considered to reflect endothelial cell dynamic balance. Miao et al revealed that lncRNA LEENE is coregulated with eNOS by the enhancer of eNOS.^30^ Upon stimulation by the enhancer of eNOS, LEENE promotes the recruitment of RNA Pol II on the promoter of eNOS to enhance the transcription of eNOS and enhance the anti‐inflammatory ability of endothelial cells. In contrast, inhibition of LEENE will restrain the expression of mRNA and protein of eNOS and increase the number of monocytes adhered to endothelial cells. These findings provide a new insight into the epigenetic regulation of gene expression of endothelial cells in atherosclerosis.

#### 4.1.2 Regulation of proliferation and apoptosis

The proliferation of vascular smooth muscle cells is another crucial process in the formation of atherosclerotic plaques. LncRNA p21, a p53‐induced lncRNA, was identified as a novel regulator of smooth muscle cell proliferation and progression of atherosclerosis. LncRNA p21 is significantly down‐regulated in atherosclerotic plaques of patients with AS and of ApoE knockout mice on a high‐fat diet.^31^ LncRNA‐p21 induces apoptosis and inhibits the proliferation of vascular smooth muscle cells. Mechanistically, lncRNA p21 enhances the transcriptional activity of p53 partly by interacting with MDM2 (mouse double microbody 2), and the activation of p53 in turn regulates cell proliferation and alleviates the progression of atherosclerosis. Ballantyne et al also identified that SMILR (muscle‐induced lncRNA enhances replication) is a promoter of vascular muscle cell proliferation, which express increasedly in unstable atherosclerotic plaques.^32^ These results indicate that lncRNA p21 and SMILR can be used as a therapeutic strategy for AS.

#### 4.1.3 Regulation of lipid metabolism

Foam cells are the characteristic pathological cells in AS and arise from phagocytosis of lipids by macrophages or vascular smooth muscle cells because of abnormal lipid metabolism. LncRNA Mexis was reported to exert great influence on lipid metabolism through regulating ABCA1, which is critical for regulation of cholesterol efflux and generation of high‐density lipoprotein (HDL). Specifically, Mexis greatly increases with the activation of liver X receptors (LXRs), which are sterol‐activated nuclear receptors regulating expression of genes involved in reverse cholesterol transport, and the increase in Mexis then augments Abca1 expression and macrophage cholesterol efflux. Further study revealed that the stable overexpression of Mexis in macrophages increases the expression of ABCA1 to eliminate cholesterol more effectively, which significantly reduces the probability of AS in mice. Mice lacking Mexis gene show tissue‐selective decreased expression of ABCA1, and the probability of vascular occlusion is twice as high as that in normal mice.^33^

### 4.2 LncRNAs and myocardial infarction

Myocardial infarction (MI), a major contributor to mortality around the globe, entails acute or persistent coronary artery ischaemia and hypoxia leading to myocardial necrosis. Early prediction and diagnosis of MI are of great importance to improve the prognosis and survival of patients. Ishii et al were the first to report that lncRNA MIAT is associated with MI.^34^ Further study testified that the genetic variation of six single‐nucleotide polymorphisms (SNP) in MIAT confers susceptibility to MI. In addition, another study showed that the expression level of MIAT in peripheral blood cells of patients with acute myocardial infarction (AMI) is regulated after AMI and associated with prognosis.^35^ Moreover, the level of MIAT correlates positively with proportions of lymphocytes and negatively with proportions of neutrophils and platelets. Overall, the variation and expression level of MIAT are of great significance in patients with MI. LncRNA HOTAIR, also was associated with MI, was a protective factor of cardiomyocytes through negative regulation of miR‐1.^36^ Compared with a healthy control group, the expression of HOTAIR was significantly decreased in the serum of mice and patients with AMI and in cardiomyocytes exposed to hypoxia, indicating that the plasma concentration of lncRNAs can be used as biomarker in the diagnosis of human AMI.

Besides being early indicators of MI, lncRNAs play a crucial role in the pathogenesis of MI by controlling autophagy, apoptosis and other processes.

#### 4.2.1 Regulation of autophagy

Autophagy is considered to be vital for renewing organelles and meeting metabolic needs under stress, both in physiological and pathological processes of the body. Although the exact effect of autophagy under different conditions has not been fully clarified, abnormal autophagy is associated with a variety of cardiovascular diseases.^37^ Wang et al found that lncRNA APF (autophagy promoting factor) increased significantly under ischaemia or reperfusion conditions and regulates autophagy by affecting the activity of downstream target ATG7 of miR‐188‐3p and miR‐188‐3p.^38^ ATG7 is an important autophagy promoting gene, and the APF‐induced inhibition of ATG‐7 restrains abnormal autophagy and exerts a protective effect on myocardium. LncRNA CAIF (cardiac autophagy inhibitor) also inhibits cardiac autophagy and reduces the damage of MI through binding to p53 and suppressing the transcription of myocardin.^39^ Knockout of the myocardin gene inhibits autophagy and alleviates cardiac ischaemia‐reperfusion injury, while the interaction between CAIF and p53 significantly blocks the expression of myocardin and exerts a protective effect. Liang also demonstrated that loss of lncRNA Mirf (myocardial infarction‐regulatory factor) alleviated cardiac injury in MI mice by regulating miR‐26a.^40^ These results show that lncRNAs may be a potential therapeutic target for the treatment of cardiovascular diseases related to autophagy.

#### 4.2.2 Regulation of apoptosis

The regulating effect of lncRNAs on apoptosis is also of great importance in alleviating MI injury. For example, knockout of lncRNA Mirt1, which is mainly expressed in cardiac fibroblasts, reduces apoptosis in cardiomyocytes and infiltration of inflammatory cells into the heart thereby improving cardiac function. The underlying mechanism involves inactivation of the NF‐ κB signal pathway induced by the lack of Mirt1.^41^ Meanwhile, inhibition of lncRNA Gpr19 and overexpression of lncRNA UVA1 are proved to attenuate cardiac injury after MI by inhibiting apoptosis via the miR‐324‐5p/Mtfr1 axis and miR‐143/MDM2/p53 axis, respectively.^42^, ^43^

#### 4.2.3 Regulation of proliferation

Loss and inability to proliferate of adult cardiomyocytes partly underlie impaired cardiac function and heart failure after cardiac injury.^44^ Although some studies regard stem cell transplantation as a promising means to replace apoptotic cardiomyocytes after injury, the low retention rate and uncontrolled differentiation of transplanted stem cells greatly hinder its application.^45^ Therefore, exploring new strategies to regenerate cardiomyocytes and reactivate proliferation ability is of crucial importance in conquering heart failure after injury,^46^ and lncRNAs are increasingly being recognized as a promising alternative. The heart‐specific lncRNA Carel suppresses cardiomyocyte mitosis by competitively suppressing endogenous miR‐296 and consequently down‐regulating the target genes miR‐296, Trp53inp1 and ITM2A. In contrast, silencing Carel significantly promotes cardiac regeneration and improves cardiac function in neonatal and adult mice after MI.^47^

Similar to Carel, lncRNA CPR (cardiomyocyte proliferation regulator) also plays an important regulatory role in the proliferation of cardiomyocyte.^48^ Knockout of CPR activates cardiomyocyte proliferation, promotes heart repair and improves cardiac function after MI, while its overexpression produces the opposite effect. A mechanistic study revealed that the negative regulatory effect of CPR in cardiac proliferation is related to its interaction with MCM3, which is the starting point of DNA replication and cell cycle process. CPR directly interacts with the CpG site of the MCM3 promoter, promotes its methylation and inhibits its expression, thereby eventually inhibiting the proliferation of cardiomyocytes.

Besides, a cardiomyocyte regeneration‐related lncRNA named CRRL was proved to suppress cardiomyocyte regeneration by binding to miR‐199a‐3p.^49^ LncRNA AZIN2‐sv regulates endogenous cardiomyocyte proliferation negatively by preventing the activation of PI3/Akt pathway.^50^ These studies provide new insights into the inability to proliferate of adult cardiomyocytes, and the theoretical basis for a lncRNAs‐based therapeutic strategy to promote cardiomyocyte regeneration and heart repair after injury.

### 4.3 LncRNAs and hypertrophy

Myocardial hypertrophy is an adaptive and protective response to overload stress; however, persistent pathological myocardial hypertrophy is often accompanied by maladaptive myocardial remodelling as manifested in various cardiovascular diseases such as hypertension and valvular disease. Pathological myocardial hypertrophy is also an independent risk factor for myocardial ischaemia, arrhythmia, chronic heart failure and sudden death.^51^ LncRNAs plays an important role in the occurrence and progression of myocardial hypertrophy. In a RNA‐Seq study, lncRNAs were dramatically differently expressed in left ventricular tissue between mice with aortic coarctation and controls.^52^ The specific roles of lncRNAs in myocardial hypertrophy are outlined below.

#### 4.3.1 Regulation of histone acetylation

Han et al identified a cardioprotective lncRNA named myosin heavy chain‐associated RNA transcripts (Mhrt), which is made up of antisense transcripts from Myh7 loci and confers protection from cardiac hypertrophy and subsequent heart failure.^53^ Specifically, under pathological stress, lncRNA Mhrt transcription is markedly inhibited by Brg1‐Hdac‐Parp chromatin repressor complex and restoring Mhrt to normal level can significantly rescue the heart from hypertrophy. Mhrt binds to the helicase domain of BRG1, which is a key histone acetylation factor, sequesters BRG1 from its genomic DNA targets, and suppresses gene acetylation regulated by BRG1 thereby eventually alleviating cardiac hypertrophy progression.

#### 4.3.2 Regulation of histone methylation

Inhibiting the expression of a heart‐rich lncRNA named CHAER in the heart dramatically reduces myocardial hypertrophy and dysfunction.^54^ LncRNA CHAER directly interacts with the catalytic subunit of histone modified complex2 (PRC2). This interaction prevents PRC2 from targeting genomic loci thereby inhibiting the methylation of H3K27 (histone3 lysine 27), which is essential for the induction of genes involved in cardiac hypertrophy. LncRNAs also positively regulates histone methylation, thereby exerting a protective effect on cardiac hypertrophy. For example, overexpression of lncRNA antihypertrophy‐related transcript (AHIT) significantly reduces stress‐induced myocardial hypertrophy in vitro.^55^ AHIT directly binds to the promoter of MEF2A, an indispensable transcriptional factor in hypertrophy, and recruits SUZ12, a core PRC2 protein. The recruited SUZ12 remarkably induces the tri‐methylation of the promoter of MEF2A thereby suppressing MEF2A transcription. A recent research identified that lncRNA H19 suppressed H3K27 tri‐methylation, which in turn reversed pathological cardiac hypertrophy.^56^ These results clarify the role of lncRNAs in cardiac hypertrophy as well as elucidate a novel target for the treatment of cardiac hypertrophy.

#### 4.3.3 Regulation of the MyD88 signal pathway

LncRNA CHRF (cardiac hypertrophy related factor) acts as an endogenous sponge RNA inhibiting the activity of miR‐489 thereby precluding suppression of miR‐489 on MyD88 and the downstream molecule NF‐κB.^57^ MyD88 is a prerequisite for Ang‐II to initiate cardiac hypertrophy, and mice with MyD88 knockout have reduced inflammatory response to Ang‐II. Therefore, CHRF‐induced activation of MyD88 would aggravate the development of cardiac hypertrophy.

#### 4.3.4 Regulation of autophagy

A recent study identified an important role for lncRNA Chast (cardiac hypertrophy associated transcript) in cardiac remodelling induced by pressure overload.^58^ The expression of Chast was up‐regulated specifically in mice with transverse artery coarctation (TAC). Human Chast homologues were also significantly up‐regulated in hypertrophic heart tissue of patients with aortic stenosis and cardiomyocytes derived from human embryonic stem cells under the stimulation of hypertrophy. LncRNA Chast inhibits the expression of autophagy regulator Plekhm1 (Pleckstrin homeodomain protein family member 1), thereby reducing autophagy and cardiomyocyte hypertrophy. Meanwhile, inhibition of Chast before or after induction of hypertrophy prevents or reduces myocardial hypertrophy, respectively, indicating that lncRNA has the potential to become a new therapeutic target.

### 4.4 LncRNAs and heart failure

Heart failure (HF) is the disturbance of cardiac circulation caused by the dysfunction of systolic and/or diastolic function of the heart and characterized by impaired cardiac structure and function. Heart failure is not an independent disease, but the final stage of the development of various heart diseases, and lncRNAs play a vital role in its occurrence and progression.^59^ A recent study analysed the transcript differences between overload‐induced heart failure and normal mice and found that 135 LncRNAs were differentially expressed.^52^ Another study more comprehensively analysing expression of mRNAs, miRNAs and lncRNAs in cardiomyocytes with HF showed that compared with the expression profiles of mRNAs or miRNAs, the expression profiles of lncRNAs are more sensitive to different aetiology of HF and the support of mechanical circulation. In other words, the expression pattern of lncRNA is aetiologically specific and sensitive to hemodynamic load conditions in HF. These results underscore the critical role of lncRNAs in HF.

#### 4.4.1 Biomarker

Although BNP and NT‐pro BNP are recognized biomarkers of HF in clinical diagnosis, their specificity and accuracy still warrant further improvement. At present, the gradually discovered strong correlation between lncRNA and HF renders lncRNA an attractive biomarker for HF. Kumarswamy et al explored circulating lncRNAs in plasma in patients with HF and found that mitochondrial lncRNA LIPCAR was down‐regulated in the early stage after MI, up‐regulated in the late stage of ventricular remodelling and further up‐regulated in patients with chronic heart failure.^60^ In addition, the level of LIPCAR significantly correlated with the risk of cardiovascular death, indicating that lncRNAs in plasma can be used for prognostication.

A recent study analysing heart tissues from patients with HF and healthy donors identified that lncRNA COL1A1, which had been associated with fibrosis, was significantly associated with the progression of HF.^61^ Additionally, the study discovered that the high level of COL1A1 in plasma was related to low survival rate of heart transplantation within 1 year after the diagnosis of HF. LncRNA H19 in plasma was identified to indicate the severity and prognosis of PAH(pulmonary arterial hypertension).^62^ These results suggest that plasma level of lncRNAs can be used as marker to distinguish the malignant process of HF.

#### 4.4.2 Regulation of cardiac fibrosis

Myocardial fibrosis is an important part of almost all forms of HF, which destroys myocardial structure and accelerates the progression from the initial heart disease to HF.^63^ Piccoli et al reported that lncRNA Meg3, mostly expressed by cardiac fibroblasts (CFs), is down‐regulated during late cardiac remodelling. Inhibition of Meg3 reduces the expression and activity of matrix metalloproteinase‐2 (MMP‐2), leading to decreased fibrosis and hypertrophy.^64^ Another cardiac fibroblast‐enriched lncRNA Whisper (Wisper 2 super‐enhancer‐associated RNA) was reported to regulate cardiac fibrosis after injury. It was demonstrated that the silencing of Wisper relieved fibrosis and cardiac dysfunction induced by MI through regulating cardiac fibroblast gene expression programmes.^65^ These results provide new insights into the mechanism of HF and help us formulate better diagnosis and treatment strategies to achieve better prognosis.

### 4.5 LncRNAs and dilated cardiomyopathy

Dilated cardiomyopathy (DCM), a primary myocardial disease of unknown origin, is characterized by impaired ventricular dilatation and systolic function usually leading to chronic heart failure. There currently are few treatments for dilated cardiomyopathy, rendering it necessary to explore the pathophysiological mechanisms underlying DCM and to elucidate new therapies. Genetic factors play an important role in the pathogenesis of DCM, and more than 20 gene mutations have been associated with DCM.^66^ LncRNAs have become new research target because of their regulatory role in gene expression.

A newly discovered lncRNA, Novlnc6, is significantly down‐regulated in DCM.^67^ Knockout of Novlnc6 in cardiomyocytes down‐regulates BMP and Nkx2‐5, which are two important regulatory factors in cardiomyocytes, suggesting that Novlnc6 might regulate protein‐coding genes in DCM. Moreover, lncRNA MIAT, which has been associated with MI as mentioned above, was significantly overexpressed in patients and mice with Chagas disease cardiomyopathy (CCC), an invasive inflammatory DCM.^68^ Furthermore, by comparing microarray analyses of DCM patients and healthy controls, Li et al found a human‐specific lncRNA DCRL, which significantly regulates DCM‐related genes in cardiomyocytes.^69^

Haas et al reported that structural genomic variant (SVs) contributed greatly to differential gene expression, including that of lncRNAs, in DCM. Among the identified 80,635 elements of the genome change caused by SVs, lncRNAs account for 3758 variations, which may underlie the basic mechanism of cardiac maladaptation in DCM.^70^ LncRNAs have the potential to treat muscle diseases by re‐establishing gene regulatory networks like miRNAs.^71^ In summary, further study on lncRNAs is of great significance to understand the pathological mechanisms underlying DCM and explore new biomarkers and gene therapy approaches.

### 4.6 LncRNAs and arrhythmia

The electrophysiological activity of the heart is disordered during arrhythmia, which is characterized by excitation conduction, membrane repolarization, automaticity, intracellular Ca^2+^ treatment, spatial heterogeneity of the above characteristics and myocardial structure (gap and scar). Arrhythmia may occur when any of these six attributes is abnormal.^20^ As detailed below, lncRNAs affect the occurrence of arrhythmia in different ways.

#### 4.6.1 Effect on Ca^2+^ transport

Magny et al firstly discovered an arrhythmia‐related lncRNA, the putative non‐coding RNA 003in2L (pncr003:2L), in *Drosophila*, which has two small open reading frames encoding small peptides containing 28 and 29 amino acids, respectively. There were more arrhythmic contractions when pncr003:2L was silenced, which resolved when the peptide chains encoded by the two small open reading frames were introduced.^72^ Further study in mammalian muscle revealed that Sln and Pln, the human homologous of pncr003:2L, regulate Ca^2+^ transport by inhibiting the activity of sarcoplasmic reticulum Ca^2+^ adenosine triphosphatase (SERCA) the function of which is to transport Ca^2+^ from the cytoplasm back to the sarcoplasmic reticulum. LncRNAs also play a negative role in regulating Ca^2+^ transport. Zhang et al found that by inhibiting the activity of SERCA2a, lncRNA ZFAS1 changed the calcium transient of cardiomyocytes, which would result in intracellular calcium overload and increase the tendency to delay late de‐grading and arrhythmias.^26^

#### 4.6.2 Effect on gap junctions

Cellular gap junctions are also an important target of lncRNAs in modulating the electrophysiological activity of the heart. Cx43 (Connexin 43) in the gap junction is abnormal in HF patients, resulting in slower conduction velocity and scattered pulse propagation, thereby increasing the risk of arrhythmias and even sudden cardiac death. LncRNA CCRR (cardiac conduction regulation RNA), a kind of antiarrhythmic lncRNA, was recently reported to improve the presence of Cx43 in gap junctions and thereby helping to maintain normal cardiac conduction.^73^ Gain or loss of function experiments also demonstrated that down‐regulation of CCRR is sufficient to induce cardiac electrophysiological disorders and arrhythmias, while overexpression of CCRR reduces conduction abnormalities and ventricular arrhythmias in mice with chronic HF. The latter evidence suggests that CCRR is a potential therapeutic target for pathological arrhythmias. Another study revealed the antiarrhythmic role of CCRR during AMI.^20^ Similar abnormalities of Cx43 in both chronic HF and AMI model suggest that CCRR may control cardiac electrical activity in patients with AMI in a similar way as in chronic HF.

#### 4.6.3 Regulation of cardiac autonomic nerve function

Atrial fibrillation (AF) is the most common arrhythmias with complex pathogenesis, high rates of morbidity and mortality and current treatments that are not optimal. Cardiac autonomic nerve function plays a crucial role in the occurrence and maintenance of AF.^74^ Considering that the cardiac fat pad is the distribution and integration centre of the inherent autonomic nerve of the heart,^75^ Wang et al analysed the fat pad of dog heart and discovered 166 down‐regulated lncRNAs and 410 up‐regulated lncRNAs.^76^ Bioinformatics analysis showed that these abnormally expressed lncRNAs are closely related to nerve growth, development, migration and neuropathy. Among these differently expressed lncRNAs, researchers confirmed that lncRNA TCONS_0032546 and lncRNA TCONS_0026102 were related to nerve remodelling by means of differential expression analysis, target gene function, bioinformatics and tissue specificity analysis. In vivo experiments also demonstrated that silencing TCONS_00032546 or TCONS_00026102 significantly shortened or prolonged atrial effective refractory period and thus induced or prevented AF, respectively. These results show that lncRNAs are involved in nerve remodelling in AF, which provides a potential therapeutic target for the prevention and treatment of AF.

#### 4.6.4 Regulation of inflammation‐related pathways

Shi et al analysed the RNA sequencing data of epicardial adipose tissue in patients with AF and discovered dysfunctional lncRNAs associated with AF pathogenesis.^77^ Among these dysfunctional lncRNAs, SNHG16 and RP11‐471B22.2 are involved in inflammation‐related pathways, suggesting that they may be related to oxidative stress, apoptosis, fibrosis and other pathological processes, thus promoting the occurrence of AF.

**FIGURE 2:**
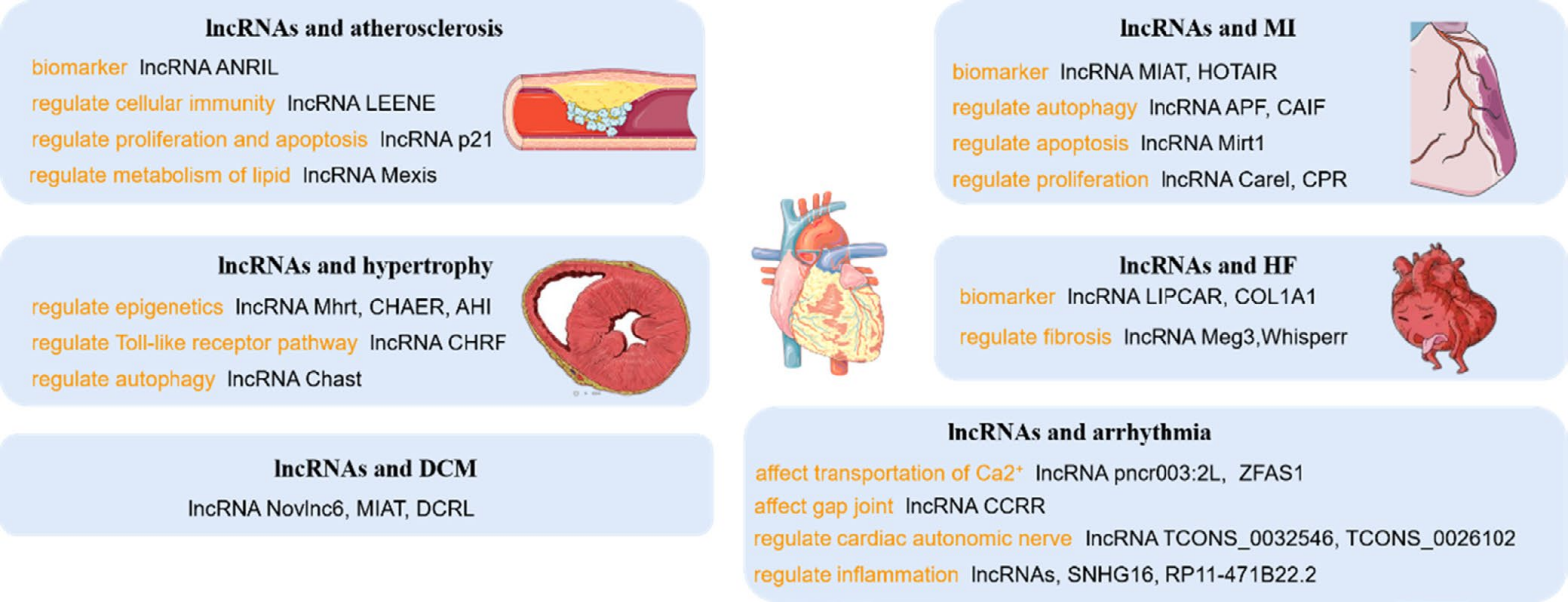
lncRNAs and cardiovascular diseases LncRNAs play different roles in diverse kinds of cardiovascular diseases. LncRNA ANRIL, MIAT and LIPCAR can respectively act as biomarker in AS, MI and HF; LncRNA LEENE regulates cellular immunity, and LncRNA Mexis can regular metabolism of lipid, which play roles in the formation and progression of AS; In MI, lncRNA APF, Mirt1 and CPR can regulate autophagy, apoptosis and proliferation of cardiomyocytes, respectively; LncRNA Mhrt and CHAER can regulate epigenetics, while lncRNA Chast can regulate autophagy to reduce dysfunction in HCM; LncRNA Meg3 and Whisper can regulate cardiac fibrosis, which eventually leads to heart failure; LncRNA ZFAS1 and CCRR can affect transportation of Ca^2+^ and gap joint, which may cause arrhythmia

LncRNA plays an important role in the occurrence and development of cardiovascular diseases such as coronary atherosclerosis, myocardial infarction, heart failure and arrhythmia and is a potential target and biomarker for prevention and treatment (see Figure 2).

## 5 CONCLUSION AND PROSPECTS

Recent studies show that lncRNAs are involved in a variety of cardiovascular diseases (see Table 1), thereby ushering a new research field and providing insight on lncRNAs as important eukaryotic transcripts. Development of large‐scale sequencing of the whole genome allowed the discovery and further research on lncRNAs and has documented their large number and variety. LncRNAs significantly regulate gene expression by various mechanisms, and it is reasonable to posit that research on lncRNAs is in its infancy and that the currently discovered regulatory roles of lncRNAs in different disease models are only the tip of the iceberg.

**Table 1:** The Roles of lncRNAs in cardiovascular disease

Disease	lncRNAs	Pathological processes	Molecular mechanism	References
Atherosclerosis	ANRIL	Biomaker		4, 29
	LEENE	Inflammation	Promote the transcription of eNOS	30
	lncRNA p21	Proliferation, apoptosis	Enhance the activity of p53	31
	SMLR	Proliferation	Modulate the expression of nearby genes such as HAS2	32
	lncRNA Mexis	Lipid metabolism	Regulate ABCA1 which is critical for regulation of cholesterol efflux and generation of HDL	33
Myocardial infarction	lncRNA MIAT	Biomaker		35
	lncRNA HOTAIR	Biomaker		36
	lncRNA APF	Autophagy	Affect the activity of downstream target ATG7	38
	LncRNA CAIF	Autophagy	Bind to p53 and suppress the transcription of myocardin	39
	lncRNA Mirf	Autophagy	Regulate Mir26a	40
	lncRNA Mirt1	Apoptosis	Regulate the NF‐κB signal pathway	41
	lncRNA Gpr19	Apoptosis	Regulate the miR‐324‐5p/Mtfr1 axis	42
	lncRNA UVA1	Apoptosis	Regulate the miR‐143/MDM2/p53 axis	43
	lncRNA Carel	Proliferation	Competitively suppress endogenous miR‐296	47
	lncRNA CPR	Proliferation	Interact with the promoter of the MCM3, promote its methylation and inhibit its expression	48
	CRRL	Proliferation	Bind to miR‐199a‐3p	49
	lncRNA AZIN2‐sv	Proliferation	Prevent the activation of PI3/Akt pathway	50
Hypertrophy	lncRNA Mhrt	Acetylation	Bind to BRG1 and suppress gene acetylation regulated by it	53
	lncRNA CHAER	Methylation	Interact with PRC2 and inhibit the methylation of H3K27	54
	lncRNA AHIT	Methylation	Bind to the promoter of MEF2A and induce the tri‐methylation of it	55
	lncRNA H19	Methylation	Suppress H3K27 tri‐methylation	56
	lncRNA CHRF	MyD88 signal pathway	Act as an endogenous sponge RNA inhibiting the activity of miR‐489	57
	lncRNA Chast	Autophagy	Inhibit the expression of autophagy regulator Plekhm1	58
Heart failure	lncRNA LIPCAR	Biomarker		60
	lncRNA COL1A1	Biomarker		61
	lncRNA H19	Biomarker		62
	lncRNA Meg3	Fibrosis	Regulate the expression and activity of MMP‐2	64
	lncRNA whisper	Fibrosis	Regulate cardiac fibroblast gene expression programmes	65
Dilated cardiomyopathy	lncRNA Novlnc6		Modulate BMP and Nkx2‐5 to regulate protein‐coding genes	67
	lncRNA DCRL		Regulates DCM‐related genes	69
Arrhythmia	lncRNA pncr003:2	Ca^2+^ transport	Inhibit the activity of SERCA	72
	lncRNA ZFAS1	Ca^2+^ transport	Inhibit the activity of SERCA2a	26
	LncRNA CCRR	Gap junctions	Improve the presence of Cx43 in gap junctions	73
	TCONS_0032546, TCONS_00026102	Autonomic nerve function	Regulate atrial effective refractory period	76
	SNHG16, RP11‐471B22.2	Inflammation‐related pathways		77

The poor stability of lncRNAs poses challenges to the research of specific biological functions and mechanisms. A newly developed method resorting to the hyperconservative elements located upstream or downstream from lncRNAs may provide new ideas for studying the cross‐species translation function of lncRNAs.^78^ Moreover, the therapeutic efficacy of lncRNAs remains promising despite their poor organ specificity. For example, although lncRNA APF is widely expressed in the whole body, it regulates autophagy and alleviates myocardial ischaemia‐reperfusion injury in mice.^38^ Because each cardiovascular disease does not occur independently, we should pay close attention to the complex coordination of lncRNAs in various cardiac cells.

With the development and application of high‐throughput lncRNAs expression analysis technology, gene chip technology and bioinformatics technology, the understanding of lncRNAs in cardiovascular system has become increasingly deep and comprehensive. These studies extend the understanding on the occurrence and development mechanism of cardiovascular diseases, as well as provide new targets for the prevention and treatment of cardiovascular diseases. LncRNAs are a new breakthrough in cardiovascular disease research with great potential to advance therapeutics.

## CONFLICT OF INTEREST

The authors declare that there is no conflict of interest regarding the publication of this article.

## AUTHOR CONTRIBUTION

**Yexian Fang:** Software (equal); Writing‐original draft (equal). **Yuerong Xu:** Software (equal); Writing‐original draft (equal). **Runze Wang:** Software (equal); Validation (equal). **Lang Hu:** Software (equal); Supervision (equal). **Dong Guo:** Software (equal); Supervision (equal). **Feng Xue:** Investigation (equal); Validation (equal). **Wangang Guo:** Investigation (equal); Validation (equal). **Dongwei Zhang:** Investigation (equal); Validation (equal). **Jianqiang Hu:** Software (equal); Supervision (equal). **Yan Li:** Writing‐review & editing (equal). **Wei Zhang:** Writing‐review & editing (equal). **Mingming Zhang:** Funding acquisition (lead); Writing‐review & editing (lead).
